# A Comparative Metabolomics Approach for Egyptian Mango Fruits Classification Based on UV and UPLC/MS and in Relation to Its Antioxidant Effect

**DOI:** 10.3390/foods11142127

**Published:** 2022-07-18

**Authors:** Mohamed A. Farag, Amr Abdelwareth, Ahmed Zayed, Tarek F. Eissa, Eric Dokalahy, Andrej Frolov, Ludger A. Wessjohann

**Affiliations:** 1 Pharmacognosy Department, College of Pharmacy, Cairo University, Kasr El Aini St., Cairo 11562, Egypt; 2 Chemistry Department, School of Sciences & Engineering, The American University in Cairo, New Cairo 11835, Egypt; amr.abdelwareth@aucegypt.edu (A.A.); erickdokalahy@aucegypt.edu (E.D.); 3 Pharmacognosy Department, College of Pharmacy, Tanta University, Elguish Street (Medical Campus), Tanta 31527, Egypt; ahmed.zayed1@pharm.tanta.edu.eg; 4 Institute of Bioprocess Engineering, Technical University of Kaiserslautern, Gottlieb-Daimler-Str. 49, 67663 Kaiserslautern, Germany; 5 Faculty of Biotechnology, October University for Modern Sciences and Arts (MSA), Giza 12451, Egypt; eissatarek@hotmail.com; 6 Department of Bioorganic Chemistry, Leibniz Institute of Plant Biochemistry, Weinberg 3, 06120 Halle (Saale), Germany; andrej.frolov@ipb-halle.de; 7 Department of Biochemistry, St. Petersburg State University, 199034 St Petersburg, Russia

**Keywords:** flavonoids, LC-MS, mango, metabolomics, multivariate data analysis, UV/Vis

## Abstract

Mango (*Mangifera indica* L.) is a tropical climacteric fruit that encompasses a myriad of metabolites mediating for its nutritive value, unique taste, flavor, and medicinal uses. Egypt is among the top mango producers worldwide, albeit little characterization has been made toward its fruits’ chemical composition. This study aims to assess metabolites difference via comparative profiling and fingerprinting of Egyptian mango in context to its cultivar (cv.) type and/or growth province. To achieve such goal, hyphenated chromatographic techniques (UPLC/MS) and UV spectroscopy were employed and coupled to multivariate data analysis for Egyptian mango fruits’ classification for the first time. UPLC/MS led to the detection of a total of 47 peaks identified based on their elution times and MS data, belonging to tannins as gallic acid esters, flavonoids, xanthones, phenolic acids and oxylipids. UV/Vis spectra of mango fruits showed similar absorption patterns mostly attributed to the phenolic metabolites, i.e., gallic acid derivatives and phenolic acids showing λ_max_ at ca. 240 and 270 nm. Modeling of both UPLC/MS and UV data sets revealed that cv. effect predominated over geographical origin in fruits segregation. Awees (AS) cv. showed the richest phenolic content and in agreement for its recognition as a premium cv. of mango in Egypt. Results of total phenolic content (TPC) assay revealed that AS was the richest in TPC at 179.1 mg GAE/g extract, while Langara from Ismailia (LI) showed the strongest antioxidant effect at 0.41 mg TE/g extract. Partial least square modeling of UV fingerprint with antioxidant action annotated gallates as potential contributor to antioxidant effect though without identification of exact moieties based on UPLC/MS. The study is considered the first-time investigation of Egyptian mango to aid unravel phytoconstituents responsible for fruits benefits using a metabolomics approach.

## 1. Introduction

Mango (*Mangifera indica* L., family Anacardiaceae) is a tropical tree native to the Indo-Burmese peninsula of Asia. However, it is cultivated currently in more than 87 tropical and subtropical countries all over the world with ca. 1000 varieties [1,2]. Based on the Food and Agriculture Organization of the United Nations (FAO) reports in 2019, India is leading mango production with 22 million tons per year, representing 43% of the total world’s production [3]. Moreover, other countries were listed as major producers, including Mexico, China, Indonesia, and Egypt. Mango can either be consumed domestically or used as an additive in the food industry owing to its unique flavor. Particularly, the Egyptian mango was introduced from Sri Lanka in 1825 [4]. However, it is now among the top 10 mango producers globally, with a total annual production of more than 600 thousand tons annually in 2019, based on the official statement issued by the Egyptian Ministry of Agriculture [4]. In addition, Egyptian mango has acquired a worthy reputation worldwide, possessing various cultivars (cvs.) of unique flavors and tastes [4,5].

Different parts of mango trees, including fruits pulp/flesh (45–65% of the whole fruit fresh weight) and peel/skin, seeds, leaves, bark, roots, and flowers, have been phytochemically investigated [2]. Mango fruit encompasses a myriad of metabolite classes ranging from low molecular and volatile molecules, primary and secondary metabolites, as well as their degradation byproducts, which differ greatly in types and concentration according to several factors, *viz.* cultivar type, geographical origin, ripening stage, and their processing method [6]. Specifically, the phytochemical composition of various mango fruits cvs. have been investigated based on ultra-performance liquid chromatography (UPLC) coupled to a photodiode array detector (PDA) and an electrospray ionization quadrupole time-of-flight tandem mass spectrometer (ESI–QTOF/MS) showing their richness in wide array of phytochemicals and essential nutrients. For instance, both macronutrients (e.g., sugars, amino acids, lipids, and organic acids) and micronutrients (e.g., vitamin A and C and minerals) were reported to be major constituents proving mango potentials to be a food of high nutritional values. Asides, phytochemicals, including polyphenols (e.g., flavonoids, xanthones, and phenolic acids), pigments (e.g., carotenoids), and volatile constituents [7]. Examples include phenolic acids such as ferulic, chlorogenic, and caffeic acids, in addition to polyphenols content, i.e., mangiferin, ellagic acid, gallotannins, and quercetin [7,8,9,10]. Such richness in potential bioactives and nutraceuticals has resulted in additional ethnopharmacological and nutritive values. Nevertheless, various cvs. of mango fruits revealed potential compositional differences both qualitatively and quantitatively, as in the case of Chinese cvs. [8].

Moreover, previous reports revealed well-documented anti-inflammatory, antioxidant, antimicrobial, gastro- and hepato-protective, immunomodulatory, and anti-hyperlipidemic effects for mango fruits [1,11,12]. Particularly, antioxidant activity has attracted special interest considering its robustness to measure and reflect an estimate of phenolic composition in fruit flesh part. Hoyos-Arbeláez et al. assessed the antioxidant activity of Hilacha cv. from a Colombian local market and showed that mango seed extract exhibited the highest antioxidant capacity (773.7 ± 7.9 µmol TEs/g DW as determined using 2,2-diphenyl-1-picrylhydrazyl (DPPH) assay), followed by peel (551.57 ± 3.05 µmol TE/g DW) and pulp (65.7 ± 2.6 µmol TE/g DW) [13]. In addition, carbohydrates such as pectin (5–11%) and cellulose (30%) content were detected in fruit peels with potential industrial and food applications [14,15].

A multiplexed approach in food metabolomics based on analytical techniques, either indirect as LC/MS or direct, including nuclear magnetic resonance (NMR) and ultraviolet-visible spectrometry (UV/Vis), has been increasingly reported in food authentication and quality control [16,17]. Particularly, LC/MS is well suited for the profiling of phytoextracts, especially after the recent development of UPLC/MS, owing to its improved peak resolution and high sensitivity. In contrast, UV/Vis spectrometry is recognized as a non-destructive, simple, cheap, and fast technique for fingerprinting rather than structural annotation of phytochemical products [18].

With the large amount of data sets generated by these techniques, multivariate data analyses (MVA) either in unsupervised, i.e., principal component analysis (PCA), or supervised mode, i.e., orthogonal partial least-squares discriminant analysis (OPLS-DA), are used for the identification of potential markers for investigated samples classification and products fingerprinting and authentication. Such an approach has increasingly been applied for the quality control of functional foods in the context of determination of freshness, geographical and genotype, processing, and or adulteration detection. Examples include our previous reports in *Hypericum perforatum* (St. John’s Wort), *Humulus lupulus* L. (hop), and *Crocus sativus* (saffron) [19,20,21]. In mango fruits, metabolomics-based phytochemical profiling was also investigated using GC/MS for the determination of various fruit-ripening practices in relation to their quality [22,23].

In the case of Egyptian mango fruits represented by its different major cvs., fewer reports have been made in the literature with regard to their phytochemical composition, except for a recent report on its aroma composed of low molecular weight volatile metabolites based on headspace solid phase microextraction (SPME) coupled with GC/MS [5], in addition to other studies for mango byproducts, i.e., seed kernel, investigating their antioxidant (e.g., increase in oxidative stability of sunflower oil) and antimicrobial (e.g., against *Escherichia coli* strain and improvement in shelf lifetime of cow milk) activities [24]. The work presented herein aimed at further dissecting mango metabolome using an untargeted UPLC/MS- and UV-Vis-based metabolomics approach for phytochemicals profiling and fingerprinting of 17 Egyptian mango cvs. (e.g., Founs, Awees, Sokary, and Zibdea) and/or growth province, i.e., Sharqia Governorate (9 samples), Alexandria desert road (Nubaria) (4 samples), and Ismailia Governorate (4 samples), comparatively with regards to its secondary metabolites’ composition. Asides, the current study attempted for larger metabolites annotation compared to previous literature, which used reference standards that were not available for all metabolites in mango fruits. The comparative metabolomics approach was coupled to MVA for Egyptian mango fruits classification is presented for the first time. Further, assessment of cvs. antioxidant activity in relation to metabolites’ fingerprint was attempted to determine whether differences in metabolites composition affect antioxidant action in Egyptian mango fruits.

## 2. Materials and Methods

### 2.1. Manog Fruit Samples

A total of 17 Egyptian mango fruit samples represented by 11 cvs. Were collected at the fully ripened stage from three different provinces in Northern Egypt, including Sharqia Governorate (9 samples/cvs.), farmlands on the Cairo-Alexandria desert road (Nubaria) (4 samples), and Ismailia Governorate (4 samples). Nubaria fruit samples have been denoted to be cultivated in a sand soil type versus muddy type in the case of Sharqia and Ismailia Governorates, Supplementary Figure S1.

These cvs. included Hendy kalb el toor, Balady Arneba, Hendy Sinara, El Saady, Jolivia, Sokary, Sokary White, Naomy, and Langara from one origin. In addition, two samples from Founs and three samples from Zibdea and Awees were collected from different origins. Details including geographical origin, cvs. details and codes used in this study are presented in Table 1.

### 2.2. Experiment and Data Processing

#### 2.2.1. Fruits Extraction for LC/MS and UV/Vis Analyses

Fruits were first manually pitted to remove seeds from fruit pulp. Samples from fruit pulp without the peel were then lyophilized using Benchtop Freeze Dryer, LYO60B-1PT, until complete dryness and finely powdered using Kenwood KHH326BK Multione Mixer 1000 W. Samples were extracted from mango fruit pulp following the protocol used previously by El-Hawary et al. [18]. In brief, 150 mg of each freeze-dried mango pulp sample was homogenized using a Turrax mixer (11,000 rpm) with 5 mL 100% methanol (MeOH) containing internal standard umbelliferone at a concentration of 10 µg/mL for five times 20 s periods including 1 min interval between each mixing. Mango fruit debris was then removed by centrifugation at 3000× *g* for 30 min. Afterward, a 500 mg C_18_ cartridge previously conditioned with 100% MeOH and Milli-Q water was used to partially purify 1 mL aliquots that were twice eluted with 3 mL MeOH. The eluents were concentrated under a nitrogen stream, where the remaining residue was resuspended in 1 mL MeOH. Finally, 2 μL were subjected to analysis in triplicates (*n* = 3) from three different collected fruits analyzed under the same conditions using the UPLC system (Dionex Ultimate 3000, Thermo Fisher Scientific, Bremen, Germany) coupled to a MicroTOF-Q hybrid quadrupole time-of-flight mass spectrometer (Bruker Daltonics, Bremen, Germany) which is equipped with an Apollo II electrospray ion source in negative ion mode. In the range of *m*/*z* 100 to 1000, the eluted metabolites were detected. Regarding the instrument column, Water Acquity HSS T3, RP-18 was applied with the following character, including 150 × 1 mm, 1.8 µm particle size, and pore size of 100 A.

Additionally, 200 µL of MeOH fruit extracts were pipetted into microplate wells (*n* = 4) of the 96-well quartz cell of the Gen 5 UV/Vis microplate reader (BioTek Instruments, Inc., Winooski, VT, USA). The absorption spectra were recorded in the range of 200–650 nm.

#### 2.2.2. Multivariate Data Analysis (MVA) of Mango Fruit Data Sets

MVA was applied to classify mango cvs in an untargeted manner and to aid in identifying markers for each cv. or origin. The normalized data matrix of UPLC/MS to the spiked umbelliferone internal standard was modeled using PCA and OPLS-DA. While PCA was used to visualize spacing between the different mango sample groups as an unsupervised model, OPLS-DA served as a discriminatory tool to identify how two mango sample sets are discriminated from each other. Variations between sample sets were demonstrated using a score plot either in PCA or OPLS-DA model using SIMCA software (version 14.1).

Likewise, UV/Vis spectral data matrix exported using excel (Excel 2016, Microsoft^®^, Redmond, WA, USA) for all samples, including their replicates, were mean-centered and pareto scaled for variables representing absorbance readings between 200 and 650 nm. The data set was then modeled, similar to UPLC/MS, using PCA and OPLS-DA models. The OPLS-DA models were calculated using the default seven-fold cross-validation method yielding acceptable R2X, R2Y, and Q2 with no negative values and values for both above 0.5. In addition, permutations tests and CV-ANOVA with a *p*-value less than 0.05 for significance were used to validate the results of the developed OPLS-DA models.

### 2.3. Determination of Total Phenolics and Antioxidant Activity

#### 2.3.1. Total Phenolics Content (TPC)

TPC was determined colorimetry in triplicates (*n* = 3) using Folin–Ciocâlteu reagent following the protocol used by El-Hawary et al. [18] and gallic acid as a quantification standard. In brief, 20 µL of each mango cultivar methanol extract (prepared in Section 2.2.1) were mixed with 100 µL of 10% Folin–Ciocâlteu reagent before incubation in a dark place for 5 min. Afterward, 80 µL of 7.5 mg/mL sodium bicarbonate was added and left again for 30 min in the dark. Finally, absorbance was recorded at 765 nm using the Gen 5 UV/Vis microplate reader (BioTek Instruments, Inc., Winooski, VT, USA). Gallic acid was used in a concentration range between 1 and 100 µg/mL for the construction of the calibration curve. The TPC was expressed as mg gallic acid equivalent/g extract (mg GAE/g extract).

#### 2.3.2. Ferric Reducing Antioxidant Power (FRAP) Assay

FRAP assay was conducted in triplicates (*n* = 3), where 175 µL of freshly prepared FRAP reagent (10 mM TPTZ (2,4,6-tripyridyl-*S*-triazine) in 40 mM in HCl (10 Mm), acetate buffer (300 mM, pH 3.6), 20 mM FeCl_3_) were mixed in a 96-well microplate with 25 μL of mango methanol extract (prepared in Section 2.2.1). The mixture was then incubated in the dark for 30 min and recorded at 593 nm afterward using the Gen 5 UV/Vis microplate reader (BioTek Instruments, Inc., Winooski, VT, USA). A Trolox and ascorbic acid (AA) solution was used to construct the assay’s calibration curves in a concentration curve of 0.01–0.1 mg/mL. The results (mean ± SD) were expressed as mg Trolox and AA equivalents per g extract as mg TE/g extract and mg AA/g extract, respectively [25].

Statistical analysis using one-way ANOVA with the aid of the Tukey method (*p* < 0.05) was applied for the classification and grouping of investigated mango cvs. based on TPC and antioxidant effect.

## 3. Results

### 3.1. Metabolites Identification Using UPLC/MS

**Figure 1:**
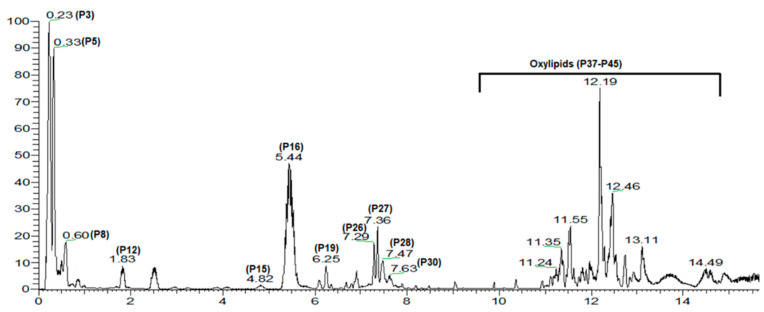
UPLC/MS total ion chromatogram (TIC) chromatogram of mango fruit methanol extract detected in negative ion mode with peaks numbering following that listed in Table 2.

Metabolites profiling of the methanol extract of mango fruit was analyzed based on UHPLC–ESI–MS operated in negative ionization mode (Figure 1). A total of 47 peaks were assigned according to their elution times and MS data, i.e., accurate molecular ion mass detected as [M-H]^−^ or as its formate adduct [M-H+ 46]^−^, and fragmentation pattern (MS^2^), in comparison with previous phytoconstituents reported in mango fruit (Table 1). The compounds belonged to organic acids, sugars, tannins as gallic acid derivatives, flavonoids, xanthones, and oxylipids, in addition to a few unknowns.

Organic acids and sugars amounted to a major class group represented by citric/isocitric acid (P2 and P5) and sucrose (P1 and P3), appearing as the most abundant peaks in mango cvs. These results are in accordance with previous data revealing that citric acid and sucrose as the predominant acid and sugar in mangoes, respectively [26]. Major secondary metabolite classes included phenolic acids, gallates/gallotannins present mostly as glycosidic conjugates.

Identification of glycosides was based on characteristic mass loss of either 132 amu (pentose), 162 amu (hexose), or both as manifested in peaks 7, 8, 11, 13, 16, and 17 belonging to glycosides of gallic, *p*-hydroxybenzoic, ferulic, sinapic, and dihydro-sinapic acids, respectively [27,28,29]. These compounds belong chemically to phenolic acids reported to contribute to mango health benefits, including antioxidant, cytotoxic, and antimicrobial effects [30].

Additionally, several polyphenols such as gallotannins were identified, including methyl gallate (P12), methyl digallate ester/isomer (P25 and P27), methyl trigallate ester (P31), pentagalloyl glucose (P26), hexagalloyl glucose (P28), and heptagalloyl glucose (P30), in accordance with the literature [26,27]. Loss of 152 amu in gallotannins aided in their identification and determination of their acylated group numbers [31,32]. Gallotannins of mangoes were reported to exhibit various effects, including antioxidant, antibacterial, and antifungal activities [26].

Moreover, flavonoid aglycones and glycosides were also detected in peaks P20, P32, P34, and P39, corresponding to quercetin-*O*-pentoside, rhamnetin-*O*-hexoside, quercetin-*O*-hexoside, and rhamnetin, respectively and showing the typical sugar losses of hexoses (−162 amu) and pentoses (−132 amu) as observed in phenolic acids. Xanthones, a unique secondary metabolite class in mango, were represented by mangiferin/isomangiferin in P16 based on its deprotonated molecular ion at *m*/*z* 421 and production at *m*/*z* 281. In the later elution range of chromatogram typical for lyophilic classes elution type, several abundant peaks were detected assigned to oxylipids, i.e., fatty acids or glycerides, and to contribute to the rich fat content in mango fruits represented by P46 rt. 12.2 min (*m*/*z* 452.32419, C_25_H_40_O_7_^−^) assigned for acylated glycerol I as the most abundant within that class.

### 3.2. UV/Vis Spectral Analysis

**Figure 2:**
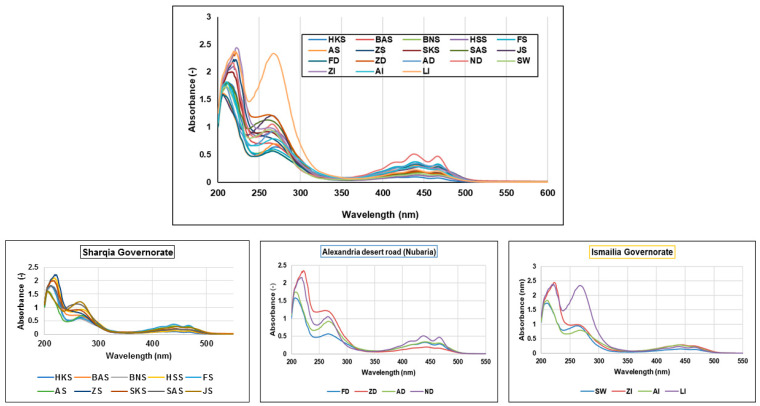
UV/Vis spectra of different mango fruits showing similar behavior mostly attributed to phenolic compounds, except that LI and ND samples showed prominent peaks of phenolic acids at ca. 270 nm and *β*-carotene between 400 and 500 nm, respectively. Three sub-figures were designed to show the fine differences between mango fruits based on geographical origin. The sample codes are listed in Table 1.

Based on the advantages of UV/Vis spectrometry, including its non-destructive, fast, and robust nature, samples were measured in parallel using the same extraction condition as explained in Section 2.2.1. UV/Vis spectra of investigated mango fruits showed a similar absorption pattern mostly attributed to phenolic compounds, including gallic acid derivatives, *p*-hydroxybenzoic acid, and catechins showing λ_max_ at ca. 240 and 270 nm [33,34], except that of ND sample, which showed few additional peaks (Figure 2). The observed difference was between 400 and 500 nm specific for carotenoids, *viz. β*-carotene [35], which may indicate its abundance in that cv. Carotenoids are known for their various bioactivities such as antioxidant, anti-inflammatory, and anti-cancer [35]. However, the presence of such marker was not revealed using LC/MS since it was performed using ESI mode, in which carotenoids are unlikely to ionize, warranting such a complementary approach of UV and UPLC/MS for mango fruits profiling.

### 3.3. UPLC/MS and UV/Vis-Based MVA of All Mango Fruit Data Sets

Considering the large number of analyzed Egyptian fruit specimens totaling 50 collected from 3 different origins, i.e., Sharqia, Ismailia, and Nubaria regions analyzed using UPLC/MS, samples were first subjected to MVA using the PCA model to assess for heterogeneity in metabolites in an untargeted manner. These samples represented 17 sample types represented by 3 biological replicates, with the PCA model score plot accounting for only 33% of the total variance as prescribed by PC1 (20.2%) and PC2 (13.0%).

**Figure 3:**
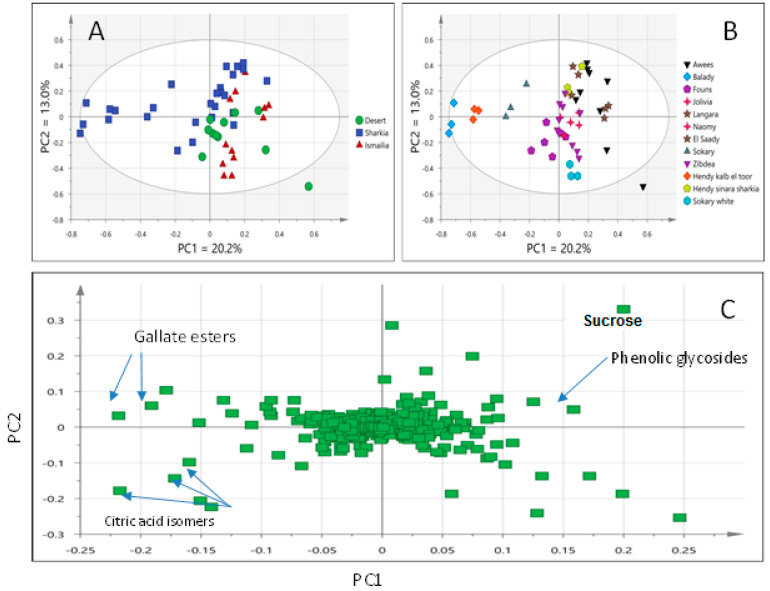
Score plot of PCA model of all mango samples UPLC/MS data classified based on geographical origin (**A**), cultivar (**B**), and corresponding loading plot (**C**).

Classification of all samples stratified based on geographical origin (Figure 3A) showed overlap between the different fruit sample subsets belonging to Sharqia, Ismailia, and desert (Nubaria) regions. Samples clustering indicated a lack of common characteristics in all mango cvs. cultivated in different regions and that higher contribution of cv.-related characteristics to the variability among fruit samples existed (Figure 3B). Examination of the loading plot revealed that variation in mostly sugars/organic acids as primary metabolites versus gallate esters as secondary metabolites accounted for fruit specimens’ segregation (Figure 3C).

Applying the same PCA model using cultivar demarked samples (Figure 3B) showed a distinct pattern. For example, cvs. Balady, Hendy kalb el toor, and Sokary cultivated in Sharqia were well spaced from other cvs., positioned to the left of other mango samples along PC1. In contrast, Awees (cultivated in different regions) and Langara (cultivated in Ismailia) cvs. were positioned to the right along PC1 (Figure 3B), suggestive that cv. effect overcomes geographical origin to a large extent in the case of mango fruit classification as analyzed using UPLC/MS. Similar findings were found in our previous work investigating volatile organic compounds in the same mango fruit data set using SPME/GC-MS [5].

Examination of the loading plot revealed that citric acid derivatives (citric acid, isocitric acid, and methyl citrate) along with gallate derivatives (e.g., methyl digallate and methyl trigallate esters) were more abundant in Balady, Hendy kalb el toor, and Sokary cvs. and accounting for its segregation. In contrast, sugars, i.e., sucrose, along with phenolic glycosides, i.e., ferulic acid, sinapic acid, and dehydro sinapic acid) were found more abundant in Awees and Langara cvs. (Figure 3C). Awees cv. is a premium mango cv. in Egypt, and its richness in sugars and phenolics might account for its improved taste and health effects compared to other cvs.

**Figure 4:**
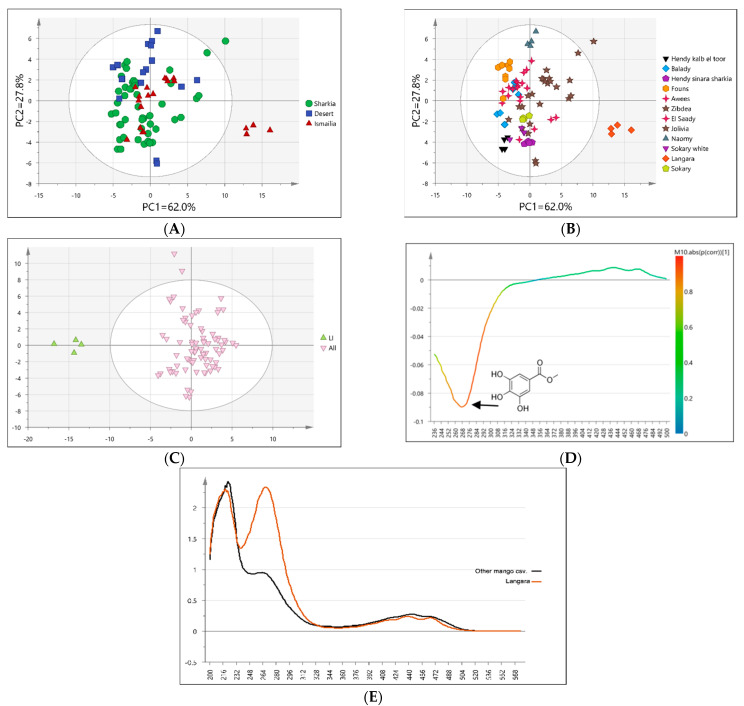
Unsupervised UV-based PCA score plot of all mango accessions classified based on geographical origin (**A**) and cultivar (**B**). Supervised OPLS-DA score plot of Langara cv versus all other Mango cvs. (**C**), corresponding S-line plot (**D**), and UV spectrum of Langara vs. other mango cvs. (**E**).

UV spectral data set of mango samples was further subjected to MVA as a direct fingerprinting tool to compare its classification potential to that of UPLC/MS, especially that phenolics likely to absorb in UV mediated for specimens’ segregation analyzed using UPLC/MS (Figure 3C). PCA model score plot accounted for 90% of the total variance as prescribed by PC1 (62.0%) and PC2 (27.8%). Classification of all samples stratified based on geographical origin (Figure 4A) showed overlap between different accessions belonging to each origin, i.e., Sharqia, Ismailia, and desert (Nubaria) regions. This indicated a lack of common characteristics in all mango cvs. cultivated in certain geographical regions, and that higher contribution of cv.-related characteristics to the variability among fruit samples (Figure 4B) and in agreement with UPLC/MS results (Figure 3 A,B). Employing the same PCA model using cv.-demarked samples (Figure 4B) showed a distinct pattern, with Langara (cultivated in Ismailia) positioned to the right along PC1 (Figure 4B) appearing as an outlier and the most different from all fruit accessions.

To confirm Langara’s unique UV fingerprint from other accessions, a supervised OPLS-DA model was attempted by modeling Langara cv. in one class group against all other mango cvs. in another class group revealing clear discrimination between Langara cvs (Figure 4C). The model was verified for its high R2x 0.835, Q^2^ 0.676 values, and *p* < 0.05 and to confirm the unsupervised PCA model score result. Further, the S-line plot of the OPLS-DA model revealed that Langara cv. exhibited wavelength distinct UV absorbance at 260–270 nm corresponding to that of gallate moiety and in accordance with UPLC/MS results. Accordingly, UV analysis could complement UPLC/MS to serve as a quality control tool for the discrimination of Langara cv. among mango accessions (Figure 4D). Examination of the UV spectrum of Langara cv. versus all other mango cvs. confirmed its unique UV spectrum (Figure 4E).

### 3.4. UPLC/MS and UV-Based Multivariate Analysis of Mango Data Subsets

Due to the low percentage of model variability encompassed by PC1 and PC2 in all samples PCA score plots (Figure 3A,B), various subsets of samples were subjected to unsupervised data analysis. Modeling was based either on different cultivars cultivated in specified geographical regions or one cultivar collected from different geographical origins.

#### 3.4.1. Awees Cultivar Classified Based on Geographical Origin

**Figure 5:**
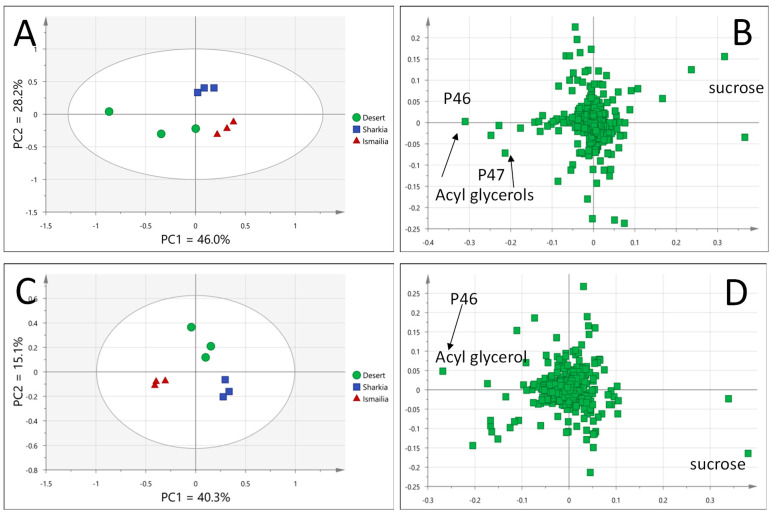
UPLC/MS PCA score plot of Awees cv. collected from different geographical origins (**A**), PCA loading plot of Awees cv. model (**B**), PCA score plot of Zibdea cv. collected from different geographical origins (**C**), PCA loading plot of Zibdea cv. model (**D**).

Awees cv. samples were obtained from different geographical regions and are considered a premium cv. in Egypt, warranting its modeling against other accessions to identify markers unique for that cv. UPLC/MS-based PCA score plot showed suitable separation of Awees cv. based on geographical origin, with PC1 and PC2 accounting for 46% and 28.3% of total model variability (74.3%), respectively. Samples from desert road farmlands (Nubaria) were positioned along PC1 to the left with negative score values, whereas samples from Ismailia and Sharqia were located in the right direction of PC1 and separated from each other along PC2, likely due to similar climatic conditions and soil type in Ismailia and Sharqia distant from desert type soil in the desert (Figure 5A). Sharqia and Ismailia are close to each other and distant from Nubaria specimens in origin.

Examination of the corresponding loading plot in the PCA model showed that acyl glycerols in P46 and P47 were more abundant in mango fruits from desert regions, while sugar at P2 was more abundant in samples from Ismailia and Sharqia regions (Figure 5B).

#### 3.4.2. Zibdea cv. Classified Based on Geographical Origin

Fruits from Zibdea cv. were likewise obtained from different geographical regions and were of interest to identify whether geographical origin affects its metabolite profile. Modeling of Zibdea cv from different origins separately using PCA showed suitable separation of samples based on geographical origin, with PC1 and PC2 accounting for 40.3% and 15.1% of the total variance (55.4%), respectively. Samples from Ismailia were positioned along PC1 to the left with negative score values, while samples from Sharqia were located in the right direction of PC1. Samples from desert regions were midway aligned on PC1 but separated from other samples along PC2 (Figure 5C). Examination of the loading plot revealed that acyl glycerol in (P46) was more abundant in fruits obtained from the Ismailia region, whereas sugar (P6) was more abundant in Sharqia and desert region (Figure 5D). These results revealed from both Awees and Zibdea cvs. suggested that primary metabolites accounted more for discriminating geographical origin within the same cv. versus phenolics, later appearing more discriminatory among different mango cvs. It should be noted that primary metabolites are less strong as markers in models considering their large variation based on several agronomic factors [36].

### 3.5. Phenolic Content in Relation to Antioxidant Activity among Mango Fruit cvs.

Previous reports showed that mango fruit pulp is rich in various phenolic constituents, including phenolic acids, flavonoids, and tannins [37]. Additionally, UPLC/MS results of the current study revealed for the enrichment of Egyptian mango cvs. in phenolic acids, flavonoids, and gallic acid derivatives (Table 2). Therefore, TPC assay was employed as a QC method to aid in the standardization of the mango cvs. cultivated in Egypt as a proximate method in quality control analysis.

Polyphenols have been well documented to exhibit free radical scavenging and metal chelation properties, including reactive oxygen species (ROS), i.e., hydroxyl, hydrogen peroxides, and superoxide radicals [38,39]. DPPH, 2,2-azino-bis (3-ethylbenzothiazoline-6-sulfonic acid) (ABTS), and FRAP are commonly employed as in vitro spectrophotometric antioxidant assays for foods [38].

In the current study, TPC was determined and related to the antioxidant activity as determined using FRAP assay (Table 3). Results showed that TPC was in the range of 179.1–26.7 mg GAE/g extract, with the highest level detected in AS versus the lowest in BNS, both collected from Sharqia province. The enrichment of phenolics in AS cv. is in accordance with modeling results (Figure 3C) and accounts for its premium type among Egyptian mango fruits. Other cvs. to encompass higher TPC were LI and SAS detected at 173.9 and 164.4 mg GAE/g extract, respectively. The previous report concerning TPC determined that mango pulp derived from different Chinese cvs. showed lower TPC values at 2.2–9.7 mg GAE/g extract, and likewise for fruits from Brazilian and Mexican cvs. [8,40]. However, such comparison may not be conclusively attributed to different factors involved as geographical origin, cv. type, and others.

With regards to the antioxidant activity of examined mango fruits cvs. (Table 3), FRAP results were expressed in both mg TE/g extract and mg AA/g extract. Results ranged from 0.41 to 0.05 mg TE/g extract and 0.08 to 0.02 mg AA/g extract, where the highest and lowest activity was found in LI and BNS, respectively. LI showed the highest total phenolics in addition to its richness in gallate, as revealed from UV fingerprinting (Figure 4E). Statistical analysis at *p* < 0.05 resulted in various groups reflecting significant differences between investigated mango cvs. (Table 3).

The antioxidant results could be correlated to TPC and also were in agreement with previous literature [37]. Regression modeling for TPC against FRAP in AA or TE equivalent produced significant models in both cases (*p* < 0.05), Supplementary Figure S2A,B). However, the quality of the model was rather low in both cases (R^2^ < 30%), which indicated that other factors contributed to the antioxidant activity other than just total phenolics content, such as total carotenoids not monitored in this assay.

### 3.6. Correlation between FRAP Assay and UV-Vis and UPLC–MS Data Sets

**Figure 6:**
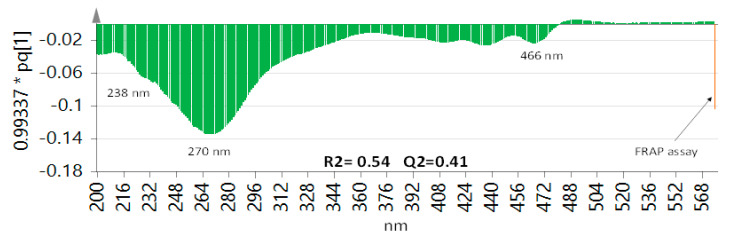
Partial least-squares (PLS) model by assigning UV peak abundance as x-variables and the corresponding FRAP results as y-variables.

A correlation between antioxidant activity and UV-Vis and UHPLC–MS data sets was attempted one at a time to assign metabolites responsible for the antioxidant activity of the different mango samples based on UV max or peaks revealed in UV and UPLC/MS data sets, respectively. Hence, a partial least-squares (PLS) model was deployed by assigning UV peak abundance as x-variables and the corresponding FRAP results as y-variables. The PLS score plot explained 54% of the total variance in Y (R^2^ = 0.54 and Q^2^ = 0.41), and as a prediction parameter explained 41%, the loading plot displaying a positive correlation with FRAP assay (Figure 6).

Investigation of variable importance in projection (VIP) enabled recognition of UV absorbance responsible for the antioxidant effects and the pinpointing of the relation between the x- and y-variables in the PLS model. The main potential UV max that had significant VIP scores included 238 and 270 nm, in addition to less contribution from that 466 nm. UV max at 270 corresponds to that of gallate moieties and is suggestive that they account for the most antioxidant effect in mango fruit pulp and much less contribution for carotenoids based on UV max at 466 nm. To identify exact gallate moieties or chemicals mediating antioxidant action in mango fruit, UPLC/MS peak abundance data set was further modeled against FRAP assay results using OPLS. A model with poor prediction power could only be retrieved and suggestive that UPLC/MS metabolite fingerprint failed to identify the exact phenolic mediating for antioxidant activity. Employment of other antioxidant models and inclusion of mango cvs. showing large differences in metabolite levels could aid in pinpointing active agents.

## 4. Conclusions

A UPLC/MS- and UV-based metabolomics approach was conducted for the characterization and discrimination of various mango cvs. cultivated in three different geographical origins in Egypt for the first time to unravel phytoconstituents responsible for fruit health benefits. A total of 47 peaks were tentatively annotated in the Egyptian mango based on UPLC/MS platform for the first time. Asides, UV/Vis spectrometry, confirmed the richness of mango fruits in phenolic metabolites. Classification of all samples stratified based on geographical origin revealed overlap between the different fruit sample subsets and suggested that a larger contribution of cv.-related characteristics to the variability among fruit samples existed compared to geographical origin. Results revealed from modeling both Awees and Zibdea cvs. collected from different origins suggested that primary metabolites account more for discriminating geographical origin than phenolics, later class appearing more discriminatory among different cultivars. The same metabolomics platform can indeed be used to assess other conditions such as climate and agricultural practices on mango fruit metabolome. Moreover, the antioxidant results were found to be correlated with TPC, especially gallate moieties, which was confirmed by the PLS loading plot, whereas other metabolites were less likely to contribute to the antioxidant activity, i.e., carotenoids. Hence, further work is required to profile the mango samples in the positive ionization mode of the UPLC/MS analysis. In addition, isolation and structural elucidation of bioactive compounds from high-quality cv. should be targeted, followed by the application of quantitative NMR (qNMR) for standardization of its extracts in the future. Further, profiling of mango fruit seed and peel as waste byproducts should now follow to aid maximize their economic value based on detailed chemical profiling using the same approach as presented in this study.

## Figures and Tables

**Table 1:** Mango fruit cvs. and their geographical locations in Egypt from which samples were collected. Mango codes used within the manuscript are listed in the table by two- to three-letter codes, while the locations of collections are shown in Supplementary Figure S1.

Geographical Origin	Cultivar (cv.)	Code
Sharqia Governorate (muddy soil)	Hendy kalb el toor	HKS
	Balady Arneba Sharqia	BNS
	Hendy Sinara Sharqia	HSS
	Founs	FS
	Awees	AS
	Zibdea	ZS
	Sokary	SKS
	El Saady	SAS
	Jolivia	JS
Alexandria desert road (Nubaria, sand soil)	Founs	FD
	Zibdea	ZD
	Awees	AD
	Naomy	ND
Ismailia Governorate (muddy soil)	Sokary White	SW
	Zibdea	ZI
	Awees	AI
	Langara	LI

**Table 2:** List of annotated metabolites in mango fruits using UPLC/MS detected in negative ionization mode and their classes.

Peak No.	Rt. (min)	[M-H]^−^	Molecular Formula	Error (ppm)	MS/MS	Identification	Class
1.	0.15	179.05582	C_6_H_11_O_6_^−^	4.5	129	Glucose	Sugar
2.	0.2	683.2238	C_24_H_43_O_22_^−^	0.3	341, 179	Unknown tetrasaccharide	Sugar
3.	0.23	191.0195	C_6_H_7_O_7_^−^	4.7	173, 111	Citric/Isocitric acid	Organic acid
4.	0.3	683.2238	C_24_H_43_O_22_^−^	0.3	341, 179	Unknown tetrasaccharide	Sugar
5.	0.33	191.0195	C_6_H_7_O_7_^−^	4.4	173, 111	Citric/Isocitric acid isomer	Organic acid
6.	0.44	341.1079	C_12_H_21_O_11_^−^	0.4	179	Sucrose	Sugar
7.	0.5	205.0349	C_7_H_9_O_7_^−^	2.8	173, 143, 111	Citric acid Methyl ester	Organic acid
8.	0.6	331.0660	C_13_H_15_O_10_^−^	0.07	169, 125	Galloylhexose	Gallotannin
9.	0.85	299.0763	C_13_H_15_O_8_^−^	0.39	239, 209, 179, 137	*p*-Hydroxybenzoic acid -*O*-hexoside	Phenolic acid
10.	0.93	381.04489	C_16_H_13_O_11_^−^		321, 263, 233	Unknown	
11.	0.96	341.1074	C_12_H_21_O_11_^−^	1.2	179	Sucrose isomer	Sugar
12.	1.83	183.0295	C_8_H_7_O_5_^−^	3.83	168, 124	Methyl gallate	Gallate
13.	1.98	355.1024	C_16_H_19_O_9_^−^	0.17	193	Ferulic acid-*O*-hexoside	Phenolic acid
14.	2.55	443.1906	C_21_H_31_O_10_^−^	1.41	425, 281, 263, 237, 219, 189, 161, 143	Unknown glycoside	
15.	4.86	355.1023	C_16_H_19_O_9_^−^	0.08	193	Ferulic acid-*O*-hexoside isomer	Phenolic acid
16.	5.62	421.07544	C_19_H_17_O_11_^−^	−2.04	281	Mangiferin/isomangiferin	Xanthone
17.	6.1	385.1856	C_19_H_29_O_8_^−^	0.38	223, 205, 161, 153	Unknown glycoside	
18.	6.2	335.0399	C_15_H_11_O_9_^−^	0.66	183	Methyl digallate ester	Gallate
19.	6.26	517.2278	C_24_H_37_O_12_^−^	0.33	385, 205, 168	Sinapic acid-*O*-hexoside-pentoside	Phenolic acid
20.	6.33	433	C_20_H_33_O_10_^−^		301	Quercetin-*O*-pentoside	Flavonoid
21.	6.36	519.2437	C_24_H_39_O_12_^−^	0.09	387, 371, 313, 218, 186, 148	Dihydrosinapic acid-*O*-hexosyl-pentoside	Phenolic acid
22.	6.68	443.1906	C_21_H_31_O_10_^−^	1.2	425, 399, 281, 263, 237, 219, 161, 143	Unknown glycoside	
23.	6.88	261	C_12_H_21_O_6_^−^		243, 201, 187	Unknown	
24.	6.91	403.1596	C_18_H_27_O_10_^−^	0.6	241, 197	Unknown glycoside	
25.	7.25	335.0399	C_15_H_11_O_9_^−^	0.48	183, 124	Methyl digallate ester isomer 1	Gallate
26.	7.29	939.1080	C_41_H_31_O_26_^−^	1.9	769, 617, 469, 393, 169	Pentagalloyl glucose	Gallotannin
27.	7.36	335.0397	C_15_H_11_O_9_^−^	0.17	183, 124	Methyl digallate ester isomer 2	Gallate
28.	7.47	1091.1172	C_48_H_35_O_30_^−^	4.3	769, 545, 469	Hexagalloyl glucose	Gallotannin
29.	7.56	545	C_24_H_17_O_15_^−^		469	Unknown malonate conjugate	
30.	7.61	1243.1279	C_55_H_39_O_34_^−^	3.05	839, 621, 545, 469	Heptagalloyl glucose	Gallotannin
31.	7.9	487.0502	C_22_H_15_O_13_^−^	1	335, 183	Methyl trigallate ester	Gallate
32.	8.10	477.23242	C_22_H_37_O_11_^−^	−1.29	315	Rhamnetin-*O*-hexoside	Flavonoid
33.	8.22	263.12802	C_15_H_19_O_4_^−^	0.89	219, 153	Unknown	
34.	8.40	463.21704	C_21_H_35_O_11_^−^	−0.752	417, 301	Quercetin-*O*-hexoside	Flavonoid
35.	8.64	245.04451	C_13_H_9_O_5_^−^	−0.235	213, 185	Unknown	
36.	8.96	327.21634	C_18_H_31_O_5_^−^	−0.796	291, 229, 211, 171	Unknown	
37.	9.07	242.17535	C_13_H_24_O_3_N^−^	1.158	225, 181	Unknown nitrogenous lipid	Oxylipid
38.	9.09	259.06033	C_14_H_11_O_5_^−^	0.888	231, 187	Unknown	
39.	9.77	315.18011	C_16_H_27_O_6_^−^	−0.33	-	Rhamnetin	Flavonoid
40.	10.10	191.10725	C_12_H_15_O_2_^−^	3.10	146, 111	Unknown	
41.	11.29	569.27112	C_39_H_37_O_8_^−^	4.36	389, 315, 253	Unknown	
42.	11.77	311.16782	C_11_H_27_O_8_^−^	−7.14	183	Unknown oxylipid	Oxylipid
43.	11.88	513.30542	C_27_H_45_O_9_^−^	−0.75	277, 253	Unknown oxylipid	Oxylipid
44.	12.06	407.21893	C_25_H_31_O_4_^−^	−6.7	153	Unknown	
45.	12.1	489.30533	C_25_H_45_O_9_^−^	−0.98	253	Unknown oxylipid	Oxylipid
46.	12.2	452.32419	C_25_H_40_O_7_^−^	0.42	255	Acyl glycerol I	Oxylipid
47.	12.3	540.36285	C_29_H_48_O_9_^−^	0.24	480	Acyl glycerol II	Oxylipid

**Table 3:** List of total phenolics content (TPC) and antioxidant effect activity using FRAP assay for investigated Egyptian mango. The results are expressed as mean ± SD (*n* = 3). The sample code is listed in Table 1.

Sample Code	TPC (mg GAE/g Extract)	FRAP (mg TE/g Extract)	FRAP (mg AA/g Extract)
LI	173.9 ± 12.8 ^a^	0.41 ± 0.1 ^a^	0.08 ± 0.0 ^a^
BNS	26.7 ± 1.3 ^i^	0.05 ± 0.0 ^h^	0.02 ± 0.0 ^h^
SS	115.2 ± 8.8 ^b,c^	0.21 ± 0.0 ^c,d^	0.05 ± 0.0 ^c,d^
ZD	129.1 ± 7.9 ^b^	0.24 ± 0.1 ^c^	0.05 ± 0.0 ^c^
BAS	73.7 ± 5.9 ^e,f,g^	0.13 ± 0.0 ^e^	0.03 ± 0.0 ^e^
HSS	60.3± 3.6 ^f,g^	0.12 ± 0.0 ^e,f^	0.03 ± 0.0 ^e,f^
ZS	104.3 ± 7.0 ^c,d^	0.2 ± 0.0 ^d^	0.05 ±0.0 ^d^
AI	74.7 ± 5.1 ^e,f^	0.12 ± 0.0 ^e,f,g^	0.03 ± 0.0 ^e,f,g^
AS	179.1 ± 10.5 ^a^	0.2 ± 0.0 ^d^	0.05 ± 0.0 ^d^
ZI	102.1 ± 7.2 ^c,d^	0.13 ± 0.0 ^e^	0.03 ± 0.0 ^e^
FS	62.2 ± 2.1 ^f,g^	0.23 ± 0.1 ^c,d^	0.05 ± 0.0 ^c,d^
ND	75.0 ± 2.6 ^e,f^	0.14 ± 0.0 ^e^	0.04 ± 0.0 ^e^
JS	58.1 ± 2.1 ^f,g^	0.12 ± 0.0 ^e,f^	0.03 ± 0.0 ^e,f^
SaS	164.4 ± 9.8 ^a^	0.09 ± 0.0 ^f,g^	0.03 ± 0.0 ^f,g^
FD	54.3 ± 3.7 ^g^	0.28 ± 0.1 ^b^	0.06 ± 0.0 ^b^
HKS	43.9± 3.2 ^h,i^	0.08 ± 0.0 ^g,h^	0.03 ± 0.0 ^g,h^
SW	100.5 ± 7.1 ^c,d^	0.12 ± 0.0 ^e,f^	0.03 ± 0.0 ^e,f^

Statistical analysis is carried out by one-way ANOVA, and the unshared small letters between groups are the significance value at *p* < 0.05.

## Data Availability

The data presented in this study are available on request from the corresponding author.

## Supplementary Materials

The following supporting information can be downloaded at: https://www.mdpi.com/article/10.3390/foods11142127/s1, Figure S1: Map of Upper Egypt showing growth provinces from which mango samples were harvested. About 17 Egyptian mango cultivars were investigated in the current study; Sharqia Governorate (9 samples), Alexandria desert road (Nubaria) (4 samples), and Ismailia Governorate (4 samples); Figure S2: Regression analysis of TPC in response to FRAP results in AA (A) or TE (B) equivalent.
