# Supplementary material for: A Comparative Metabolomics Approach for Egyptian Mango Fruits Classification Based on UV and UPLC/MS and in Relation to Its Antioxidant Effect
Source: Foods. 2022 Jul 18;11(14):2127. doi: 10.3390/foods11142127 (PMC9318453; doi:10.3390/foods11142127)
Supplement: Supplementary file 1 [file foods-11-02127-s001.zip › foods-1816146-supplementary.pdf]

A Comparative Metabolomics Approach for Egyptian Mango Fruits Classification Based on UV and UPLC/MS and in Relation to Its Antioxidant Effect

Supplementary Figures

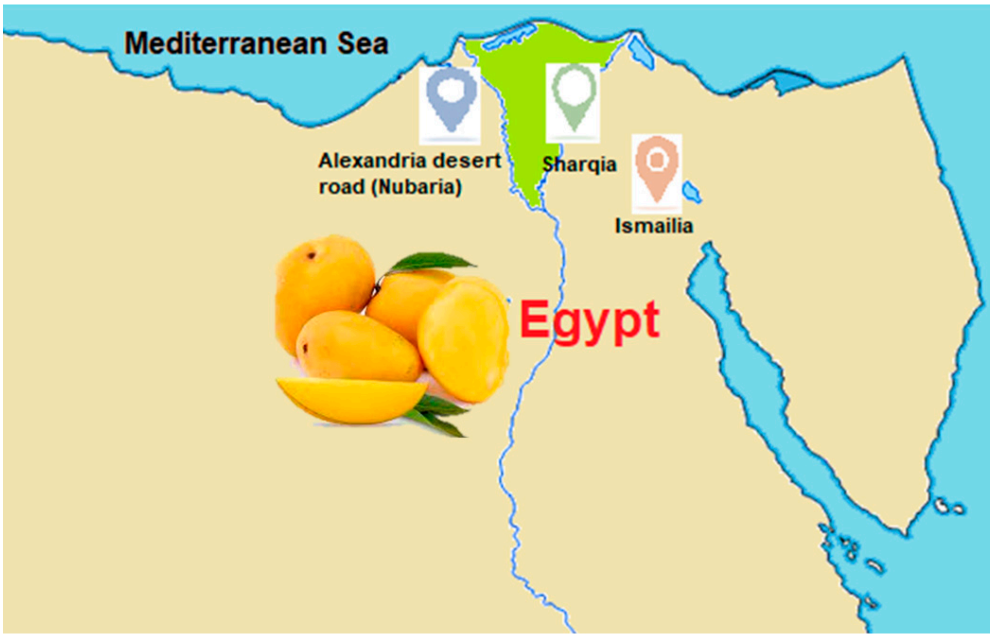

Suppl. Figure S1: Map of Upper Egypt showing growth provinces from which mango samples were harvested. About 17 Egyptian mango cultivar were investigated in the current study; Sharqia Governorate (9 samples), Alexandria desert road (Nubaria) (4 samples), and Ismailia Governorate (4 samples).

A

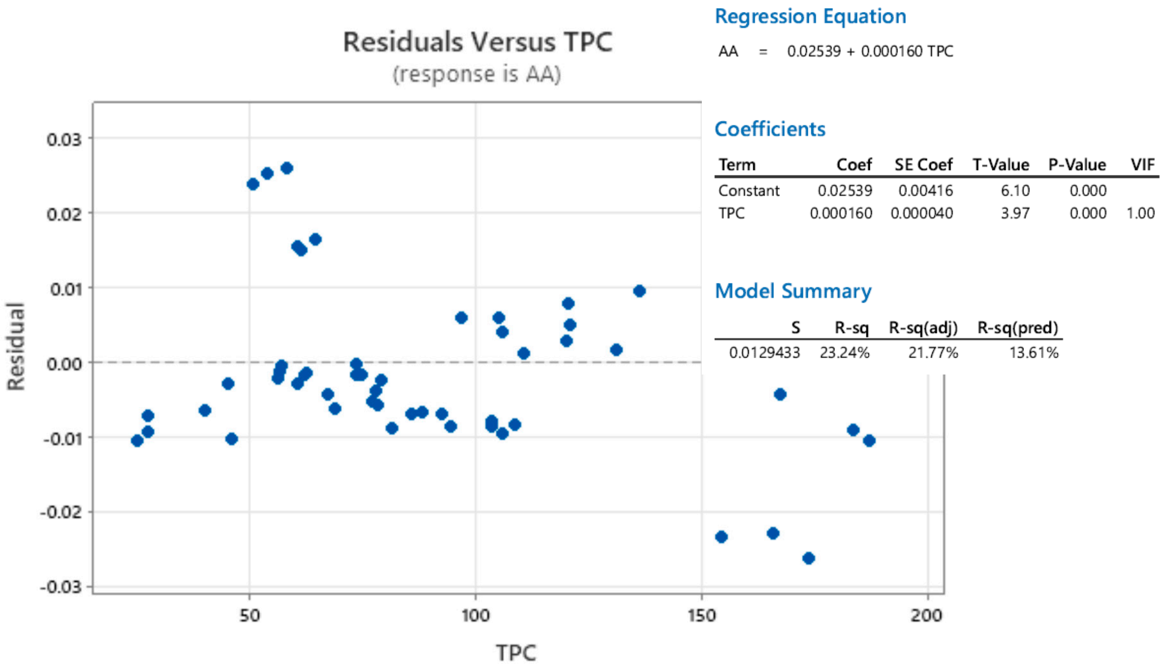

B

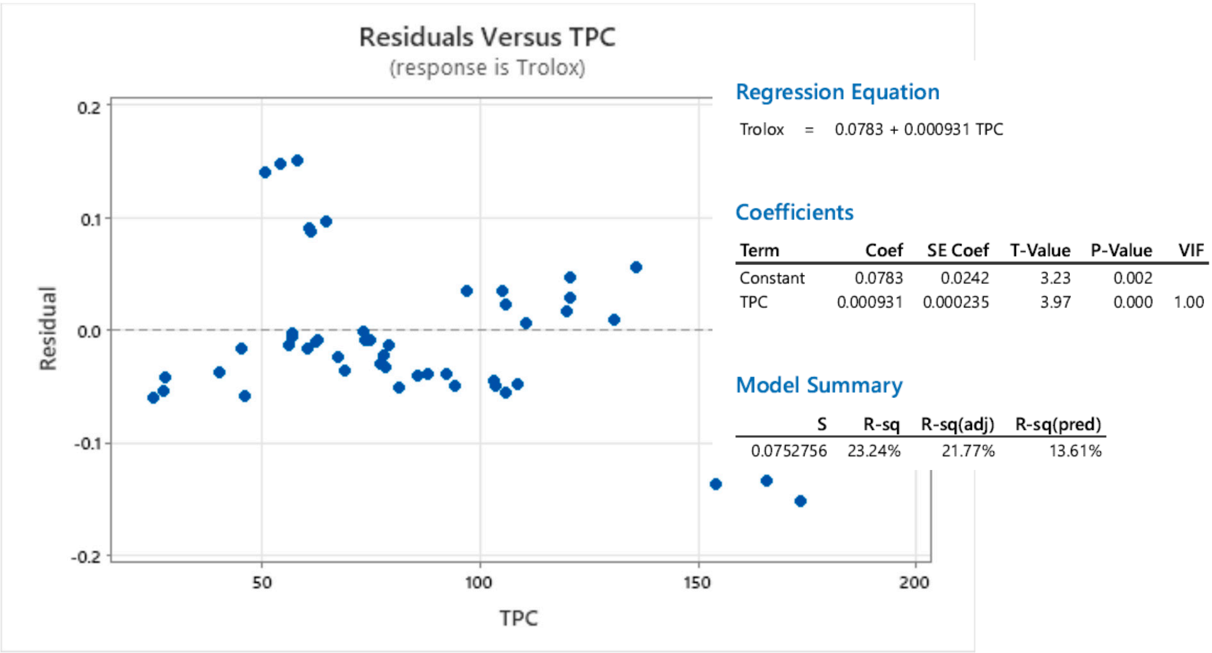

Suppl. Figure S2: Regression analysis of TPC in a response to FRAP results in AA (A) or TE (B) equivalent.
